# Anterior Subcutaneous versus Submuscular Transposition of the Ulnar Nerve for Cubital Tunnel Syndrome: A Systematic Review and Meta-Analysis

**DOI:** 10.1371/journal.pone.0130843

**Published:** 2015-06-26

**Authors:** Chun-Hua Liu, Chang-Xian Chen, Jie Xu, Han-Long Wang, Xiao-Bin Ke, Zhi-Yong Zhuang, Zhan-Long Lai, Zhi-Qiang Wu, Qin Lin

**Affiliations:** 1 Department of Orthopaedic Surgery, Quanzhou Orthopedic-Traumatological Hospital, Fujian University of Traditional Chinese Medical, Quanzhou, Fujian Province, China; 2 Department of Orthopaedic Surgery, Shengli Hospital of Fujian Medical University, Fuzhou, Fujian Province, China; 3 Department of Orthopaedic Surgery, Fuzhou Second Hospital of Xiamen University, Xiamen, Fujian Province, China; UCLA, UNITED STATES

## Abstract

**Objective:**

To pool reliable evidences for the optimum anterior transposition technique in the treatment of cubital tunnel syndrome by comparing the clinical efficacy of subcutaneous and submuscular anterior ulnar nerve transposition.

**Methods:**

A comprehensive search was conducted in PubMed MEDLINE, Cochrane Library, EMBASE, Web of Science, OVID AMED, EBSCO and potentially relevant surgical archives. Risk of bias of each included studies was evaluated according to Cochrane Handbook for Systematic Reviews of Interventions. The risk ratio (RR) and 95% confidence intervals (CI) were calculated for the clinical improvement in function compared to baseline. Heterogeneity was assessed across studies, and subgroup analysis was also performed based on the study type and follow-up duration.

**Results:**

Three studies with a total of 352 participants were identified, and the clinically relevant improvement was used as the primary outcomes. Our meta-analysis revealed that no significant difference was observed between two comparison groups in terms of postoperative clinical improvement in those studies (RR 1.04, 95% CI 0.86 to 1.25, P = 0.72). Meanwhile, subgroup analyses by study type and follow-up duration revealed the consistent results with the overall estimate. Additionally, the pre- and postoperative motor nerve conduction velocities were reported in two studies with a total of 326 patients, but we could not perform a meta-analysis because of the lack of concrete numerical value in one study. The quality of evidence for clinical improvement was ‘low’ or ‘moderate’ on the basis of GRADE approach.

**Conclusions:**

Based on small numbers of studies with relatively poor methodological quality, the limited evidence is insufficient to identify the optimum anterior transposition technique in the treatment of cubital tunnel syndrome. The results of the present study suggest that anterior subcutaneous and submuscular transposition might be equally effective in patients with ulnar neuropathy at the elbow. Therefore, more high-quality randomized controlled trials with standardized clinical improvement metrics are required to further clarify this topic and to provide reproducible pre- and postoperative objective outcomes.

## Introduction

Cubital tunnel syndrome, also called ulnar neuropathy at the elbow, is referred as the second most common entrapment neuropathy of the peripheral nerves after carpal tunnel syndrome [1, 2]. It predominantly affects the region innervated by ulnar nerve, which is characterized by pain, paraesthesias or anaesthesia, and weakness or atrophy of ulnar nerve innervated muscles. Men have about twice the mean annual incidence of morbidity of women, with estimates of both being affected almost twenty-five cases per 100,000 person-years [3].

Transpositional surgical treatments of cubital tunnel syndrome, including subcutaneous, intramuscular and submuscular [4], remain controversial, which comes from the diverging results for each of the therapeutic modality. Those who prefer anterior subcutaneous transposition claim that it produces less postoperative pain with earlier mobilization and the reduction of tension on the nerve [5, 6]. Those who advocate for anterior submuscular transposition are concerned with the new location of ulnar nerve that has a healthy vascular bed and is well protected by soft tissue [7–9]. Moreover, submuscular transposition, based on the histological study using the rat model, displayed less perineural scar tissue and healthier axons when compared to subcutaneous transposition [10].

Therefore, it is uncertain whether submusclar when compared to subcutaneous produces better clinical improvement. The reliable evidence in favor of one of two surgical treatments remains lack. Controversy exists among hand surgeons when concerning the optimum anterior transposition technique in the treatment of cubital tunnel syndrome. The objective of this systematic and meta-analysis was to pool the reliable evidences to determine which anterior transposition technique is optimum for cubital tunnel syndrome. The present study examines the evidence from randomized controlled trials (RCTs) or quasi-RCTs, includes the estimated zones of representation of approximate clinically equivalent effect sizes, and incorporates the GRADE (Grading of Recommendations Assessment, Development, and Evaluation) approach [11] to evaluate the overall quality of the evidences for the eligibility studies.

## Methods

### Protocol registration

We developed a protocol for review in advance, which registered in the PROSPERO database (protocol registration no. CRD42014015653), and followed the preferred reporting items for systematic reviews and meta-analyses (PRISMA) guidelines (S1 Table).

### Data source and search strategy

Six public databases (PubMed MEDLINE, Cochrane Library, EMBASE, Web of Science, OVID AMED and EBSCO) were searched by CHL and XBK from the inception of the databases to December 2014 without linguistic restriction. Additionally, the archives of abstracts or grey literatures were searched from the Journal of Hand Surgery, the American Society for Surgery of the Hand (ASSH), the American Association of Hand Surgeons (AAHS) and International Clinical Trials Registry Platform.

Search terms included “cubital /elbow tunnel syndrome,” “ulnar neuropathy,” “ulnar nerve compression /entrapment,” “ulnar nerve compression syndrome,” “ulnar neuropathy,” “ulnar nerve,” “subcutaneous” and “submuscular” combined with “randomized, controlled trial.” Two investigators (CHL and XBK) independently reviewed all title, abstracts, and full text of articles which might meet the inclusion criteria. Meanwhile, a comprehensive search of references from retrieved articles and relevant reviews.

### Study eligibility criteria

Studies based on all of the following criteria were selected: (1) RCTs using a truly randomized or quasi-randomized allocation of treatment were included. (2) The target participants consisted of patients who presented with primary cubital tunnel syndrome or primary ulnar neuropathy at the elbow. (3) The intervention group was anterior subcutaneous ulnar nerve transposition; (4) The comparison group was anterior submuscular ulnar nerve transposition (whether original or modified); (5) The outcomes were postoperative clinical and/ or electrodiagnostic variable defined as “improved” versus “not improved.” (6) The study described a follow-up duration of at least 12 months.

Studies were excluded if they described 1 of these conditions: (1) patient population was mixed with compressive neuropathy of ulnar nerve at another site; (2) patients diagnosed with polyneuropathy, brachial plexus injury or a general systemic disease capable of causing a non-compressive ulnar neuropathy; and (3) study was review, case report, letters or conferences.

### Assessment of risk of bias

The risk of bias of each included studies was independently evaluated by two investigators in order to assess the methodological quality of each study according to Cochrane Handbook for Systematic Reviews of Interventions. Seven domains were evaluated in each included studies: random sequence generation, allocation concealment, blinding of participants and outcome assessors, incomplete outcome data, selective outcome reporting, other risk of bias. We judged the risk of bias as ‘‘low risk”, ‘‘unclear risk” or ‘‘high risk”.

### Data extraction

According to the standard protocol, data were independently extracted by two investigators (CHL and XBK) based on the following items: (1) General information of studies included author, year of publication, country, study type. (2) Baseline characteristics of participants such as sample size, age, gender, intervention and follow-up data. (3) Primary outcomes, which were regarded as clinical improvement in function compared to baseline. (4) Secondary outcomes, consisting of adverse events, change from baseline of the cross-sectional area (CSA), motor conduction velocity (MCV), sensory conduction velocity (SCV) and neural action potential (NAP). Disagreements and differences between the investigators were resolved by consensus with all co-authors to come to an agreement. If necessary, authors of each eligible study were also contacted by e-mail to provide further information.

### Data analysis

A meta-analysis was performed using the software Review Manager 5.3.5 (Cochrane Collaboration, http://tech.cochrane.org/revman/download). For binary outcomes, the risk ratio (RR) and 95% confidence intervals (CI) were calculated, while mean difference (MD) and associated 95%CI were calculated for continuous outcomes. If outcome measurements in included studies were not conducted on the same scale, we used standardized mean difference (SMD) and 95% CI for continuous outcomes. The level of statistically significance was set at P-value<0.05. Heterogeneity among the included studies was assessed using Cochrane Handbook's Q test and I^2^ statistics [12, 13]. A P<0.05 or I^2^>50% was considered significant heterogeneity. The meta-analysis was applied by using the fixed-effect model if there was no significant heterogeneity(p≥0.05, I^2^≤50%). Otherwise, the random effect model was used or the possible reasons were explored for the significant heterogeneity (P<0.05, I^2^>50%). When data could not be collected for performing a meta-analysis, the data from these studies were evaluated as descriptive data and still considered in the results of the review.

Subgroup analysis based on study of type and duration of follow-up was then performed comparing RCT to quasi-RCT and 1 year to 2 years. The sensitivity analysis was also conducted by sequential omission of each study in turn to test the stability and strength of pooled results.

### GRADE quality assessment

GRADE quality assessment which has been increasingly adopted by many health research organizations was performed using the software GRADEprofiler 3.6 (Cochrane Collaboration, http://tech.cochrane.org/revman/other-resources/gradepro/download). Since data from RCTs were considered high-quality evidence, two investigators rated down the quality of evidence only by one for each following item: risk of bias, inconsistency, imprecision, indirectness and publication bias. Disagreements and discrepancies between the investigators were resolved by consensus with all co-authors to come to an agreement.

## Results

### Search results

**Fig 1:**
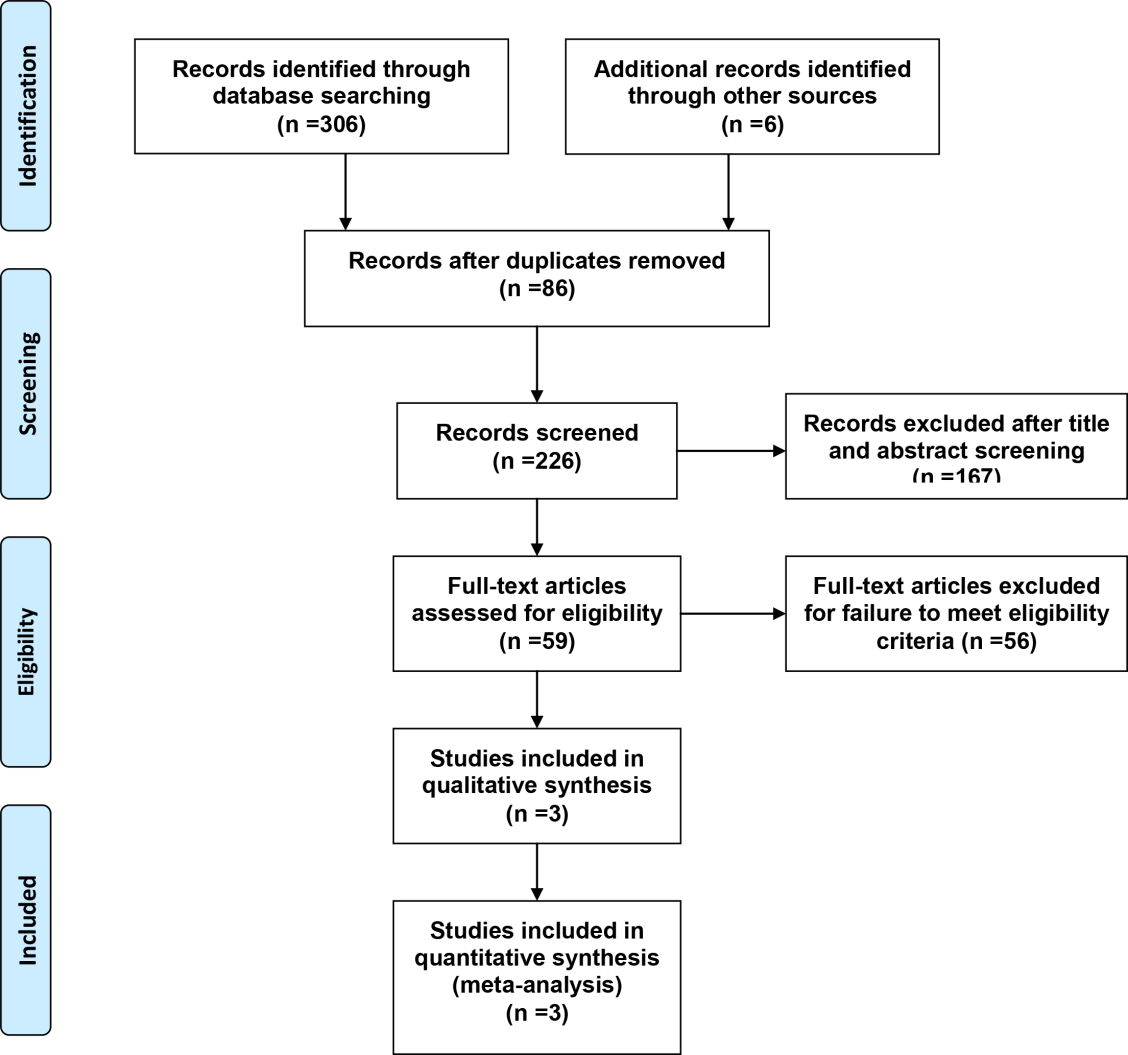
Review flow diagram.

A flow diagram that described the details of literature search was presented in Fig 1. A total of 312 potentially relevant literatures (192 from PubMed MEDLINE, 21 from Cochrane Library, 33 from EMBASE, 41 from Web of Science, 12 from OVID AMED, 7 from EBSCO and 6 from other) were identified in our initial electronic search. After removal of duplicated records, 226 literatures were remained. Then we excluded 167 inappropriate literatures by scanning the titles and abstracts. After this, the full text of remaining 59 articles were obtained and assessed for eligibility. 56 of them were further excluded for failure to meet the predefined standard protocol (S2 Table). Finally, two RCTs and one quasi-RCT were selected and analyzed in our study.

### Study characteristics

The summarized characteristics of the three studies included in the present study were presented in Table 1. These studies including 2 RCTs [14, 15] and 1 quasi-RCT [16] were published from 2009 to 2012. Among them, in one studies [16] submuscular technique of ulnar nerve was done in the original operative procedure (13 participants), whereas in the other two study [14, 15] the modified submuscular transposition was used (163 participants). The studies were conducted in Sweden [16], China [14] and Iran [15]. Data from a total of 352 patients (ranging from 26 to 378, only 1 study enrolled≥100 participants) were collected of whom 176 received subcutaneous transposition and 176 received submuscular transposition. The clinical improvement in function compared to baseline was evaluated based on different criteria in included studies. The average follow-up duration of the trails ranged from 12 to 24 months.

**Table 1:** Characteristics of the included studies.

Author	Year	Country	Study type	Subcutaneous			Submuscular			Evaluation of Procedure
				n, M/F	Mean Age (y)	Follow-up (y)	n, M/F	Mean Age (y)	Follow-up (y)	
Jaddue	2009	Sweden	quasi-RCT	13,10/3	34	1	13,10/3	34	1	Improvement or not improvement
Zarezadeh	2012	Iran	RCT	24,13/11	47.58±12.1	1	24,14/10	47.41±12.2	1	Improvement or not improvement
										Electrophysiological test
Zhong	2011	China	RCT	139	NA(32–66)	2	139	NA(32–66)	2	Improvement or not improvement
										Electrophysiological test
										Ultrasound test

RCTs: randomized controlled trials; NA: not available; M/F: man/female; y: year.

### Risk of bias in included studies

**Fig 2:**
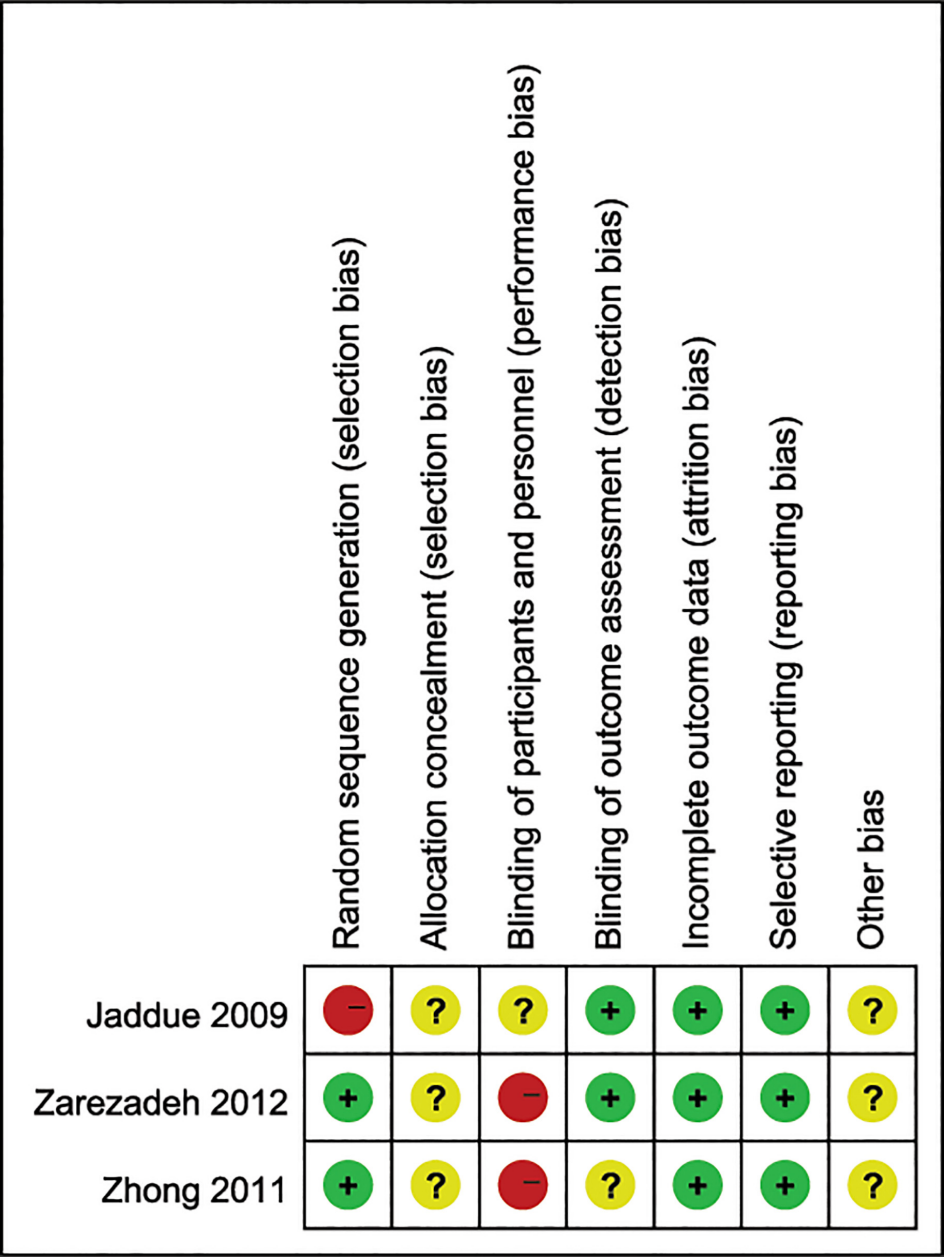
Risk of bias summary: This risk of bias tool incorporates the assessment of randomization (sequence generation and allocation concealment), blinding (participants and outcome assessors), incomplete outcome data, selective outcome reporting and other risk of bias. The items were judged as “low risk”, “unclear risk” or “high risk”.

**Fig 3:**
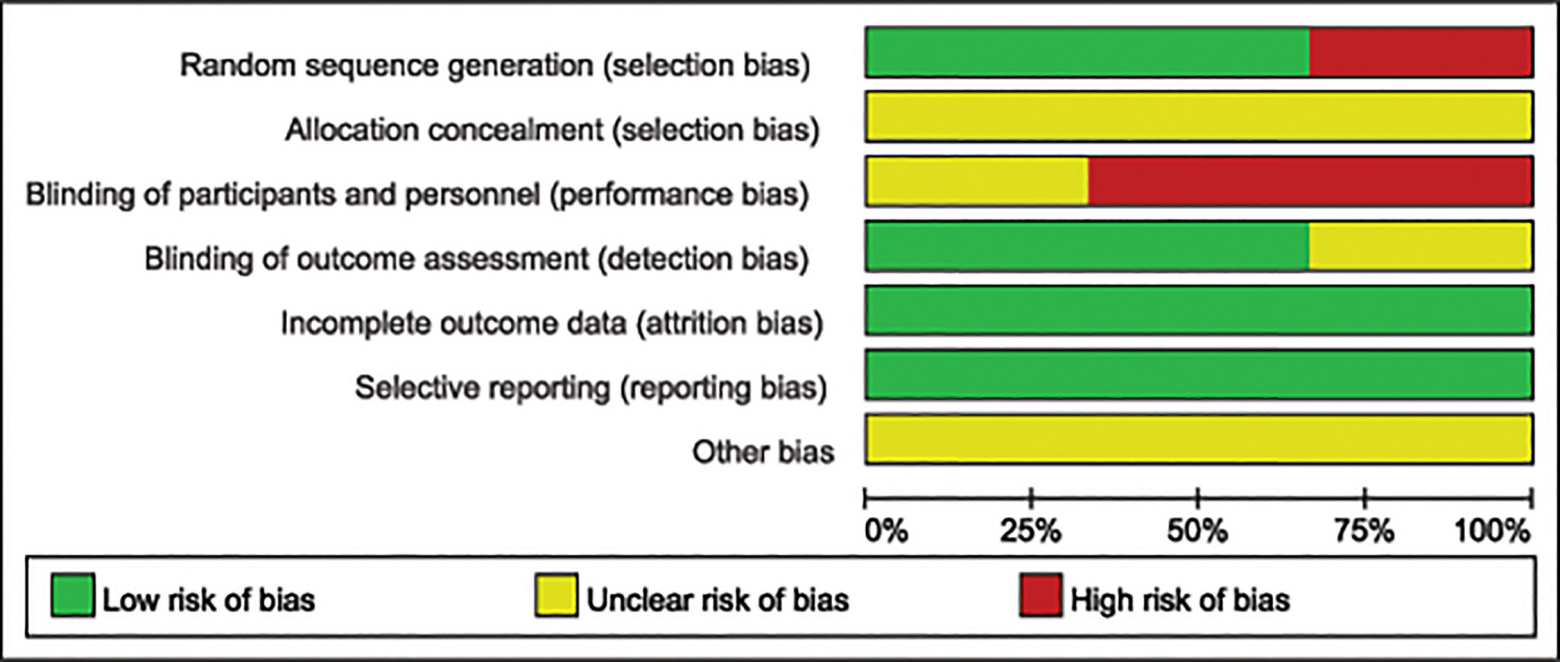
Risk of bias graph: Each risk of bias assessment was presented as the percentage across all the included studies, which indicates the proportion of different levels of risk of bias for each item.

The methodological quality of each included study has been described in Fig 2 and the judgment of “Risk of bias graph” regarding each risk of bias assessment was presented as percentages across all the three included studies in Fig 3. Among the three included studies, two RCTs [14, 15] described adequate methods of randomization, which a computer-generated list was used to randomize the participants in Zhong’s study [14] while Zarezadeh’s study [15] was based on a random table numbers. The participants of the quasi-RCT [16] were randomly assigned by age (2 years margin) and gender. Since no concrete allocation concealment method was described in three included studies [14–16], we described these studies as unclear of allocation concealment. It was not clear whether participants were blinded to the operation in Jaddue’s studies [16], and they were not blinded in two [14, 15] of the three included studies. In two studies [15, 16] all participants were evaluated by the same independent assessors while the other one [14] did not. In these studies no participant was lost to follow-up, so we regarded the included studies as low risk of incomplete outcome data. We also considered all of these studies as low risk of selective outcome reporting for they described complete outcomes in detail. Other potential sources of bias were unclear in 3 included studies since none of the studies mentioned whether or not they had raised funding in support of their research.

### Effects of interventions

#### Clinical improvement assessment

All of included studies reported proportion of participants with a clinically relevant improvement in function compared to baseline.

The clinical improvement measures differed in the three studies. Jaddue [16] used the Bishop rating system [17] after operation, which assessed subjective and objective parameters: subjective satisfaction, severity of residual symptoms (evaluated by pain, parasthesia, weakness, clumsiness), work status, leisure activity, grip strength and sensibility (static two point discrimination). Zhong [14] evaluated the clinical improvement postoperatively by including a combination of clinical presentation and physical findings (measurement of sensorimotor function). Zarezadeh [15] used Visual Analogue Scale [18], the Yale sensory scale [19], the Medical Research Council [20] and author-generated clinical scales as a way to evaluate pain, sensation, muscle strength and muscle atrophy respectively. To limit the potential source of bias when using the different assessment of clinical improvement, we reviewed the individual studies for the number of patients who improved or did not improve with each surgical treatment, and converted it into the binary categories of improvement or not improvement for this meta-analysis.

**Fig 4:**
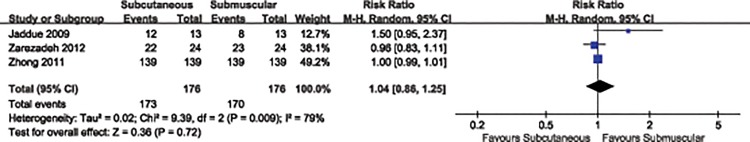
Forest plot of comparison: 1 Clinical effect of subcutaneous versus submuscular, outcome: 1.1 Proportion of patients with clinical improvement in function compared to baseline.

We found clinical improvement compared to baseline in 98.3% of the patients treated with subcutaneous transposition and in 96.7% of those treated with submuscular transposition. No significant difference was observed in postoperative clinical improvement between two treatment groups (RR 1.04, 95% CI 0.86 to 1.25, P = 0.72). (Analysis 1.1, Fig 4). A random-effects model was applied because statistical evidence of heterogeneity was found (P = 0.009, I^2^ = 79%). Sensitivity analysis revealed that heterogeneity may be attributed to the inclusion of the study reported by Jaddue [16] et al, in which participant populations compared to remaining studies were relatively small. Eliminating this study from the analysis showed a substantially reduced heterogeneity (P = 0.95, I^2^ = 1%). But we did not drop this study because only three included studies provided the clinically relevant improvement information and moderate methodological quality of the evidence.

**Fig 5:**
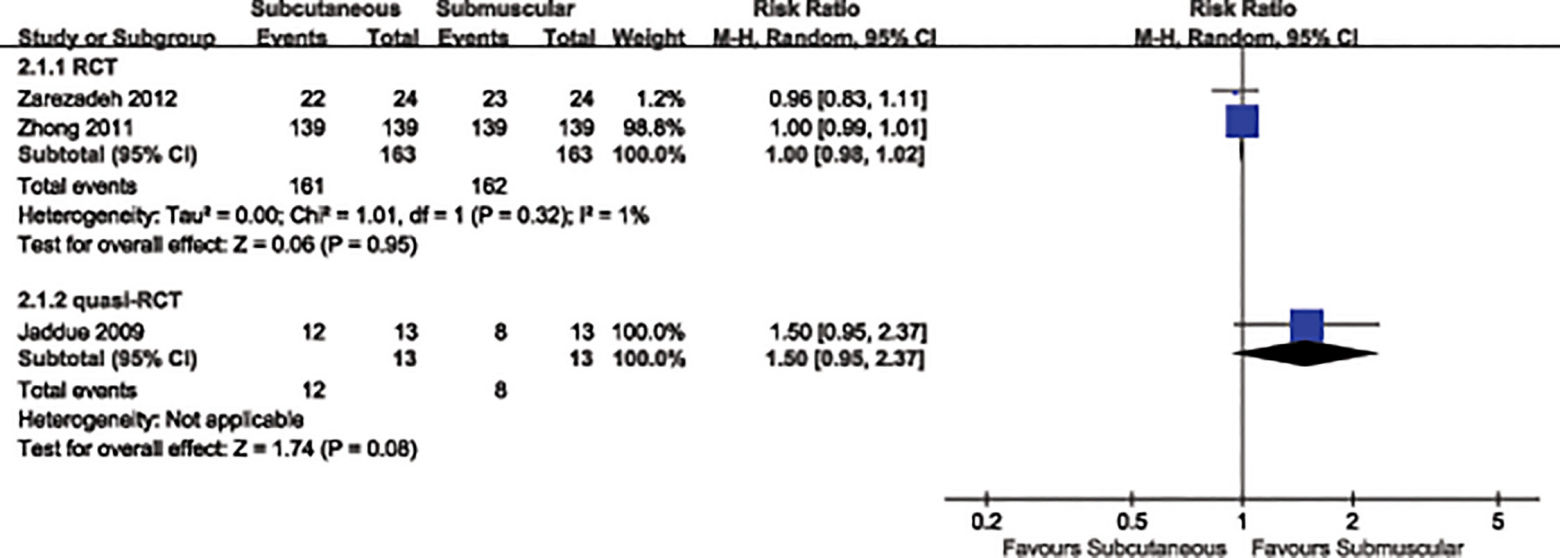
Forest plot of comparison: 2 study of subgroup, outcome: 2.1 study of type.

**Fig 6:**
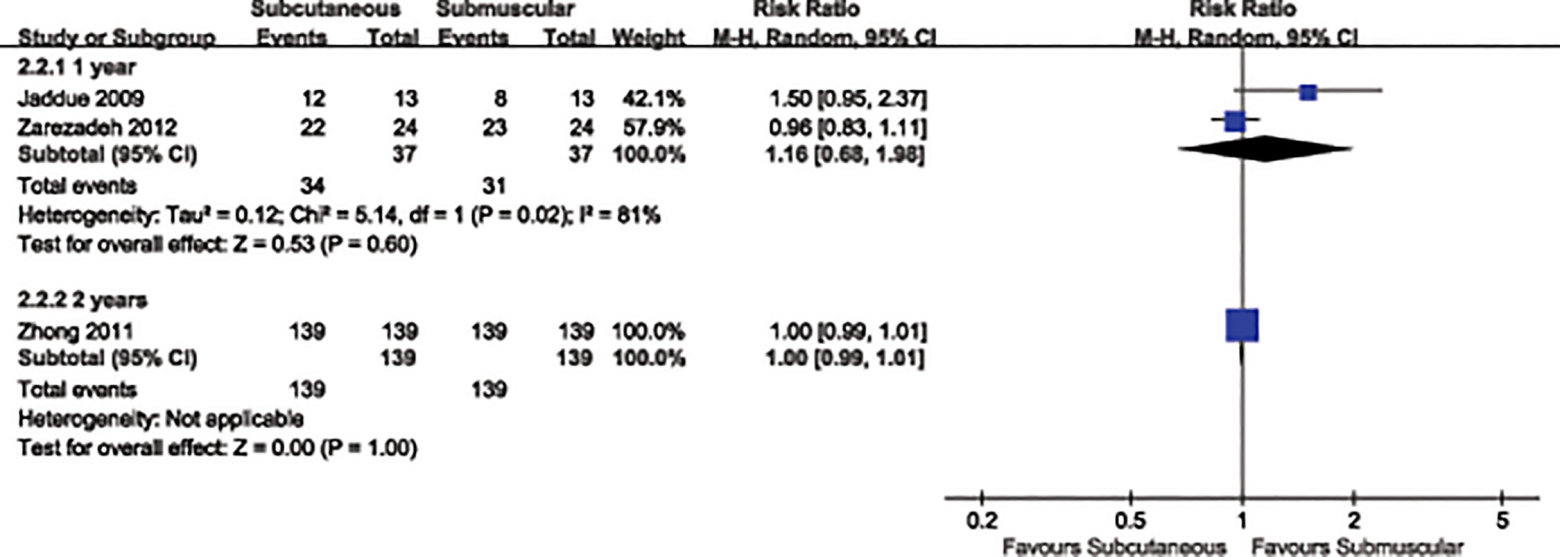
Forest plot of comparison: 2 study of subgroup, outcome: 2.2 duration of follow-up.

We then conducted two subgroup analyses for the clinical efficacy of anterior transposition of the ulnar nerve by type of study (RCT *vs*. quasi-RCT) and duration of follow-up (1 year vs. 2 years). In the subgroup analyses by study of type (RCT: RR 1.00, 95% CI 0.98 to 1.02, P = 0.95; quasi-RCT: RR 1.50, 95% CI 0.95to 2.37, P = 0.08) (Analysis 2.1, Fig 5) and duration of follow-up (1 year: RR 1.16, 95% CI 0.68 to 1.98, P = 0.6; 2 years: RR 1.00, 95% CI 0.99 to 1.01, P = 1.00) (Analysis 2.2, Fig 6), the results were in accordance with the overall estimate (Table 2).

**Table 2:** Results of subgroup analysis.

Outcome or Subgroup	Studies	Participants	Statistical Method	Effect Estimate
2.1 Study type	3		Risk Ratio (M-H, Random, 95% CI)	
2.1.1 RCT	2	326	Risk Ratio (M-H, Random, 95% CI)	1.00 [0.98, 1.02]
2.1.2 quasi-RCT	1	26	Risk Ratio (M-H, Random, 95% CI)	1.50 [0.95, 2.37]
2.2 Follow-up duration	3		Risk Ratio (M-H, Random, 95% CI)	
2.2.1 1 year	2	74	Risk Ratio (M-H, Random, 95% CI)	1.16 [0.68, 1.98]
2.2.2 2 years	1	278	Risk Ratio (M-H, Random, 95% CI)	1.00 [0.99, 1.01]

RCTs: randomized controlled trials.

#### Electrodiagnostic assessment

The pre- and postoperative motor nerve conduction velocities were reported in two studies [14, 15] with a total of 326 patients. In order to assess the clinical efficacy derived from operation in Zhong’s study [14], all patients were divided into three grades of ulnar neuropathy according to the severity of the neurological signs [21] at the time of operation: patients with mild lesions, but without detectable motor weakness, were McGowan grades I; patients with moderate lesions were McGowan grades II; patients with severe lesions that occurred marked paralysis of the ulnar intrinsic muscles were McGowan grades III.

Zhong reported measurements of postoperative MCV, SCV and NAP of the ulnar nerve at the elbow [14]. In this study, postoperative MCV, SCV and NAP were significantly better than before the operation (P < 0.05). What’s more, it was found that patients with McGowan grade II and III showed significantly greater improvements in MCV, SCV and NAP, in which the changes from baseline in submuscular group were better than in subcutaneous group, where statistically significant differences were demonstrated between MCV (r = –0.832, P<0.01), SCV (r = –0.825, P<0.01), and NAP (r = –0.862, P<0.01), while those with McGowan grades I showed no significant differences between two groups. Therefore, it may show the potential to detect a treatment effect in favor of MCV, SCV and NAP in patients with McGowan grade II and III.

To prevent bias, all participants in Zarezadeh’s study [15] underwent double-blind nerve conduction studies, conducted by the same neurophysiologists according to a standard protocol, without concrete numerical value. We sent an e-mail to the author for the original raw data, but no responses were received.

#### Ultrasound assessment

Ultrasound test was reported in only one study [14]. In this study, High-resolution ultrasound detection of postoperative CSA of the ulnar nerve was performed using the envelopment method [22–24], within one day of the electrophysiological tests.

Postoperative CSA demonstrated significantly greater improvements than before the operation (P<0.05). For McGowan grades I patients, there was no significant difference in CSA between two groups. For McGowan grades II and III, submuscular group showed significantly greater improvements in CSA than subcutaneous group.

#### Adverse events

Postoperative wound infection was reported in only one study [16]. In this study, submuscular transposition of ulnar nerve was associated with a higher number of wound infections (1/13, 7%) (one wound infection in the submuscular transposition group, zero wound infection in the subcutaneous transposition group). However, the evidence of wound infection regarding whether subcutaneous group is superior to submuscular group remains insufficient, owning to relatively small sample size of this study.

## Discussions

### Summary of main results

This systematic review and meta-analysis summarizes the results of 3 RCTs about the clinical efficacy of subcutaneous versus submuscular anterior ulnar nerve transposition for the treatment of cubital tunnel syndrome. Studies [14, 15] investigating the comparison of anterior subcutaneous transposition and modified submuscular transposition were included for this review. The available evidence in the present study suggests that anterior subcutaneous and submuscular transposition might be equally effective in the treatment of ulnar neuropathy at the elbow, because we found no statistically significant difference between two comparison groups in terms of postoperative clinical improvement compared to baseline (RR 1.04, 95% CI 0.86 to 1.25). This is true when identifying only three eligible studies or when combining two real RCTs and one relative under-representation of RCTs. Furthermore, we were not adequately powered to identify whether the detectable difference in proportion of patients with clinical improvement in subcutaneous group (98.3%) versus in submusclar group (96.7%) was actually statistically significant. With these in mind, small numbers of eligible studies and the low or moderate quality of these studies don’t allow us to reach reliable conclusions.

### Overall completeness and applicability of evidence

We identified only three studies, enrolling 352 participants, that clinical practice on the basis of varied outcomes definitely differed from population to population, and from centre to centre.

Although the preoperative status of participants among the included studies may have varied, we specified the clinical outcomes as improved or not improved regardless of whatever tool was used, the intention of which was to reduce the potential source of bias according to the predefined standard protocol. Similarly, there is controversy whether the status of participants before surgery affects the eventual postoperative outcome. however, because of inconsistency in reporting of preoperative status among studies, it was not possible to stratify for this variable that would provide useful information from the inclusion of representative studies [25]. In addition, the only three studies included, with similar interventions, small numbers of participants, limited objective information about clinical improvement in function, provide inadequate evidence that is relevant to the areas of clinical application.

### Quality of the evidence

**Fig 7:**
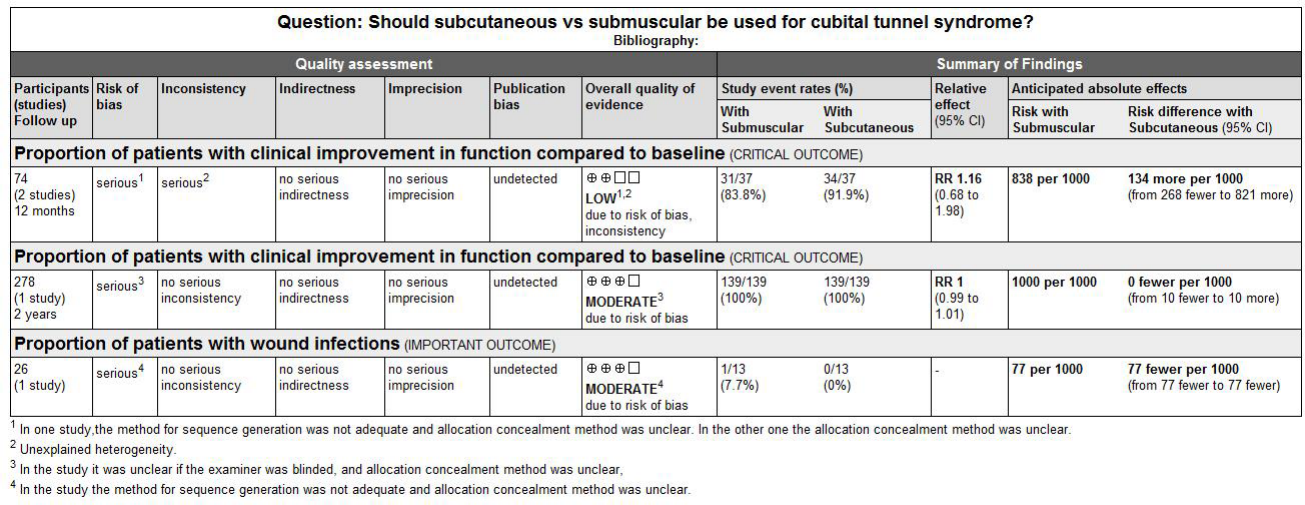
Grade profile for subcutaneous vs. submuscular for cubital tunnel syndrome.

The quality of the evidences for the primary outcomes assessed by GRADE approach was low or moderate in the present study, as shown in Fig 7. All the included studies were RCTs using a truly randomized or quasi-randomized allocation which were substantially less prone to selective bias. The method for sequence generation was adequate in two of the three RCTs included in our meta-analysis. All degrees of severity of symptoms with clinical and electrodiagnostic evidence of ulnar nerve impairment were considered. All participants were followed up for at least 12 months after operation, which showed a low risk of attrition bias.

However, some limitations in the present study should also be noted: Firstly, there was no a clear attempt in methods of randomization and allocation concealment in one study (in other two studies no concrete allocation concealment). Blinding of participants in two studies was inadequate (in one study it was unclear whether the participants were blinded). Secondly, there were only three RCTs with relatively poor methodological quality included in our meta-analysis and the efficacy of our result was relatively low considering that the quality of evidence for the primary outcomes was ‘low’ or ‘moderate’ based on GRADE approach. Thirdly, the assessment of clinical improvement in function compared to baseline was different in the three included studies, resulting in a low reliability of the results in our meta-analysis. In addition, some unpublished studies might not be included, which would lead to nonpublication bias; in the meantime, the lack of high quality of evidence limits us to further investigate the heterogeneity of the studies.

### Agreements and disagreements with other studies or reviews

To the best of our knowledge, this is the first systematic review and meta-analysis to evaluate the clinical efficacy of subcutaneous versus submuscular anterior ulnar nerve transposition for the treatment of cubital tunnel syndrome. In some previous meta-analyses [26–29] on the therapeutic management of ulnar neuropathy at the elbow, the author investigated the comparison of simple decompression and decompression with anterior transposition (subcutaneous or submuscular). And the author concluded that no significantly statistical difference in clinical outcomes between two surgical treatments was observed, but rather a trend toward less complication with simple decompression of the ulnar nerve as opposed to anterior transposition in Chen’s study [29]. A similar comparison was used by Bartels [30] and Mowlavi [31] but in these studies the authors introduced therapeutic modalities including simple decompression, anterior transposition and medial epicondylectomy. The majority of these reports analyzed the clinical outcomes as binary outcomes, but Zlowodzki [26] defined the clinical scores as continuous outcomes and used standardized mean difference (SMD). Whereas we converted the clinical outcomes into the binary categories of improved or not improved according to the registered protocol, regardless of whatever tool was used among studies. In review [27, 29], retrospective studies were also considered together in the meta-analysis, which raised the possibility of selection bias, while our study was limited to RCT or quasi-RCT.

## Authors’ Conclusions

The quality of available evidence for the primary outcomes varied from ‘low’ to ‘moderate’, and our main findings largely rely on the outcomes data from the few studies with low patient numbers. The limited evidence is insufficient to identify the optimum anterior transposition technique in the treatment of cubital tunnel syndrome. The results of the present study suggest that anterior subcutaneous and submuscular transposition might be equally effective in patients with ulnar neuropathy at the elbow. Thus, it is urgent to conduct RCT level I on the therapeutic management of cubital tunnel syndrome. Future investigation in this area should include high level of scientific evidence RCTs with standardized clinical improvement metrics to evaluate the effectiveness of two surgical options. These RCTs should be sufficiently powered to further clarify this topic and to provide reproducible pre- and postoperative objective outcomes.

## Supporting Information

S1 Table PRISMA Checklist. (DOC)

S2 Table Lists of full-text excluded articles and reasons for exclusion. (DOC)
