# Supplementary material for: Anterior Subcutaneous versus Submuscular Transposition of the Ulnar Nerve for Cubital Tunnel Syndrome: A Systematic Review and Meta-Analysis
Source: PLoS One. 2015 Jun 26;10(6):e0130843. doi: 10.1371/journal.pone.0130843 (PMC4482721; doi:10.1371/journal.pone.0130843)
Supplement: S2 Table — (DOC) [file pone.0130843.s002.doc]

**S2 Table. Lists of full-text excluded articles and reasons for exclusion.**

| Numbers | Excluded articles | Reasons for exclusion |
| --- | --- | --- |
| 1 | Clinical Results Versus Subjective Improvement With Anterior Transposition in Cubital Tunnel Syndrome | Observational study |
| 2 | Comparative Clinical Outcomes of Submuscular and Subcutaneous Transposition of the Ulnar Nerve for Cubital Tunnel Syndrome | Observational study |
| 3 | Cubital tunnel syndrome comparative results of a multicenter study of 4 surgical techniques with a mean follow-up of 92 months | Observational study |
| 4 | Subcutaneous or submuscular anterior transposition of the ulnar nerve | Observational study |
| 5 | Submuscular Versus Subcutaneous Anterior Ulnar Nerve Transposition A Rat Histologic Study | Animal study |
| 6 | [Predictors of functional outcome change 18 months after anterior ulnar nerve transposition](http://www.ncbi.nlm.nih.gov.proxy.cc.uic.edu/pubmed/22289242) | Uncontrolled |
| 7 | The results of transposition of the ulnar nerve for traumatic ulnar neuritis | Uncontrolled |
| 8 | Comparison of Transposition Techniques to Reduce Gap Associated With High Ulnar Nerve Lesions | Cadaver study |
| 9 | Surgical Outcomes of Cubital Tunnel Syndrome in Pediatric and Adolescent Patients Level 4 Evidence | Uncontrolled |
| 10 | Functional outcome of anterior transposition of the vascularized ulnar nerve for cubital tunnel syndrome | Uncontrolled |
| 11 | Anterior intramuscular transposition of the ulnar nerve for cubital tunnel syndrome | Uncontrolled |
| 12 | Comparative study of surgical treatment of ulnar nerve compression at the elbow | Uncontrolled |
| 13 | Ulnar nerve decompression by transposing the nerve and Z-lengthening the flexor-pronator mass: clinical outcome | Uncontrolled |
| 14 | Modiﬁed Intramuscular Transposition of the Ulnar Nerve | Uncontrolled |
| 15 | Comparison of Transposition Techniques to Reduce Gap Associated With High Ulnar Nerve Lesions | Uncontrolled |
| 16 | Use of a Pedicled Adipose Flap as a Sling for Anterior Subcutaneous Transposition of the Ulnar Nerve | Uncontrolled |
| 17 | [Comparative outcomes of ulnar nerve transposition versus neurolysis in patients with entrapment neuropathy at the cubital tunnel: a 20-year analysis.](http://www.ncbi.nlm.nih.gov.www.remote.uwosh.edu/pubmed/24292808) | Uncontrolled |
| 18 | [Ulnar nerve transposition using a mini-invasive approach: case series of 30 patients](http://www.ncbi.nlm.nih.gov.www.remote.uwosh.edu/pubmed/22869908) | Uncontrolled |
| 19 | Treatment for ulnar neuropathy at the elbow | Review |
| 20 | Effectiveness comparison between two different methods of anterior transposition of theulnar nerve in treatment of cubital tunnel syndrome | Observational study |
| 21 | Efficacy comparison between anterior subcutaneous and submuscular transposition of ulnar nerve to treat cubital tunnel syndrome | Observational study |
| 22 | Comparative study of surgical treatment of ulnar nerve compression at the elbow | Uncontrolled |
| 23 | Assessment of the effects of surgical treatment options for cubital tunnel syndrome on the ulnar nerve by USG and EMG | Uncontrolled |
| 24 | Anterior transposition compared with simple decompression for treatment of cubital tunnel syndrome. A meta-analysis of randomized, controlled trials | Review |
| 25 | The surgical treatment of cubital tunnel syndrome: a decision analysis | Review |
| 26 | Simple decompression versus anterior subcutaneous and submuscular transposition of the ulnar nerve for cubital tunnel syndrome: a meta-analysis | Review |
| 27 | [Ulnar nerve entrapment neuropathy at the elbow: decisional algorithm and surgical considerations.](http://www.ncbi.nlm.nih.gov.proxy.cc.uic.edu/pubmed/19266129) | Review |
| 28 | The surgical treatment of cubital tunnel syndrome: a decision analysis | Review |
| 29 | Surgical treatment for ulnar nerve entrapment at the elbow | Uncontrolled |
| 30 | Functional outcome following anterior submuscular transposition of the ulnar nerve with V-Y lengthening of the flexor-pronator origin | Uncontrolled |
| 31 | The management of cubital tunnel syndrome: a meta-analysis of clinical studies | Review |
| 32 | Surgical management of ulnar nerve compression at the elbow: an analysis of the literature | Review |
| 33 | Ulnar nerve decompression by transposing the nerve and Z-lengthening the flexor-pronator mass: clinical outcome | Uncontrolled |
| 34 | Clinical and electroneurographic results following surgical treatment of the ulnar nerve sulcus syndrome | Uncontrolled |
| 35 | Effect of submuscular versus intramuscular placement of ulnar nerve: experimental model in the primate | Uncontrolled |
| 36 | Minimum 6-year follow-up after ulnar nerve decompression and submuscular transposition for primary entrapment | Uncontrolled |
| 37 | The treatment of the cubital tunnel syndrome | Observational study |
| 38 | [Cubital tunnel syndrome a review and management guidelines](https://www.embase.com/search/results?subaction=viewrecord&rid=10&page=1&id=L361741668) | Review |
| 39 | [Simple decompression versus anterior submuscular transposition of the ulnar nerve in severe cubital tunnel syndrome: A prospective randomized study](https://www.embase.com/search/results?subaction=viewrecord&rid=2&page=2&id=L40053989) | Uncontrolled |
| 40 | Clinical efficacy of simple decompression versus anterior transposition of the ulnar nerve for the treatment of cubital tunnel syndrome A meta-analysis | Review |
| 41 | Comparative outcomes of ulnar nerve transposition versus neurolysis in patients with entrapment neuropathy at the cubital tunnel: a 20-year analysis | Uncontrolled |
| 42 | Comparison of the effects of subcutaneous anterior transposition and in situ decompression on the histologic and electrophysiologic properties of the ulnar nerve: an experimental study in a rabbit model | Uncontrolled |
| 43 | Subcutaneous anterior transposition of the ulnar nerve in cubital tunnel syndrome | Uncontrolled |
| 44 | Ulnar nerve transposition using a mini-invasive approach: case series of 30 patients | Uncontrolled |
| 45 | Cubital tunnel syndrome - a review and management guidelines | Review |
| 46 | Submuscular transposition of the ulnar nerve for the treatment of cubital tunnel syndrome | Uncontrolled |
| 47 | Anterior subcutaneous transposition of the ulnar nerve | Uncontrolled |
| 48 | Cubital tunnel syndrome--simple nerve decompression or decompression with subcutaneous anterior transposition? | Uncontrolled |
| 49 | Results of the musculofascial lengthening technique for submuscular transposition of the ulnar nerve at the elbow | Uncontrolled |
| 50 | Anterior submuscular transposition of the ulnar nerve. For post-operative focal neuropathy at the elbow | Uncontrolled |
| 51 | [Stabilized subcutaneous ulnar nerve transposition with immediate range of motion. Long-term follow-up](http://www.ncbi.nlm.nih.gov.proxy.cc.uic.edu/pubmed/11097442) | Uncontrolled |
| 52 | Ulnar nerve elongation and excursion in the cubital tunnel after decompression and anterior transposition | Uncontrolled |
| 53 | Subcutaneous anterior transposition of the ulnar nerve for failed decompression of cubital tunnel syndrome | Uncontrolled |
| 54 | Cubital tunnel syndrome: a retrospective review of 55 subcutaneous transpositions with minimum 3-year follow-up | Uncontrolled |
| 55 | Treatment of ulnar neuropathy at the elbow: cost-utility analysis | Review |
| 56 | Anterior submuscular transposition | Uncontrolled |
